# Direct quantitative identification of the “surface *trans*-effect”†
†Electronic supplementary information (ESI) available. See DOI: 10.1039/c6sc01677d


**DOI:** 10.1039/c6sc01677d

**Published:** 2016-06-09

**Authors:** Peter S. Deimel, Reda M. Bababrik, Bin Wang, Phil J. Blowey, Luke A. Rochford, Pardeep K. Thakur, Tien-Lin Lee, Marie-Laure Bocquet, Johannes V. Barth, D. Phil Woodruff, David A. Duncan, Francesco Allegretti

**Affiliations:** a Physics Department E20 , Technical University of Munich , 85748 Garching , Germany . Email: francesco.allegretti@tum.de; b Center for Interfacial Reaction Engineering , School of Chemical, Biological and Materials Engineering , The University of Oklahoma , Norman , 73019-1004 Oklahoma , USA; c Diamond Light Source , Harwell Science and Innovation Campus , Didcot , OX11 0QX , UK . Email: david.duncan@diamond.ac.uk; d Department of Physics , University of Warwick , Coventry , CV4 7AL , UK; e Department of Chemistry , University of Warwick , Coventry , CV4 7AL , UK; f ENS – Department of Chemistry , PSL Research University , CNRS UMR 8640 PASTEUR , 75005 Paris , France

## Abstract

The strong parallels between coordination chemistry and adsorption on metal surfaces, with molecules and ligands forming local bonds to individual atoms within a metal surface, have been established over many years of study. The recently proposed “surface *trans*-effect” (STE) appears to be a further manifestation of this analogous behaviour, but so far the true nature of the modified molecule–metal surface bonding has been unclear. The STE could play an important role in determining the reactivities of surface-supported metal–organic complexes, influencing the design of systems for future applications. However, the current understanding of this effect is incomplete and lacks reliable structural parameters with which to benchmark theoretical calculations. Using X-ray standing waves, we demonstrate that ligation of ammonia and water to iron phthalocyanine (FePc) on Ag(111) increases the adsorption height of the central Fe atom; dispersion corrected density functional theory calculations accurately model this structural effect. The calculated charge redistribution in the FePc/H_2_O electronic structure induced by adsorption shows an accumulation of charge along the σ-bonding direction between the surface, the Fe atom and the water molecule, similar to the redistribution caused by ammonia. This apparent σ-donor nature of the observed STE on Ag(111) is shown to involve bonding to the delocalised metal surface electrons rather than local bonding to one or more surface atoms, thus indicating that this is a true surface *trans*-effect.

## Abstract 2

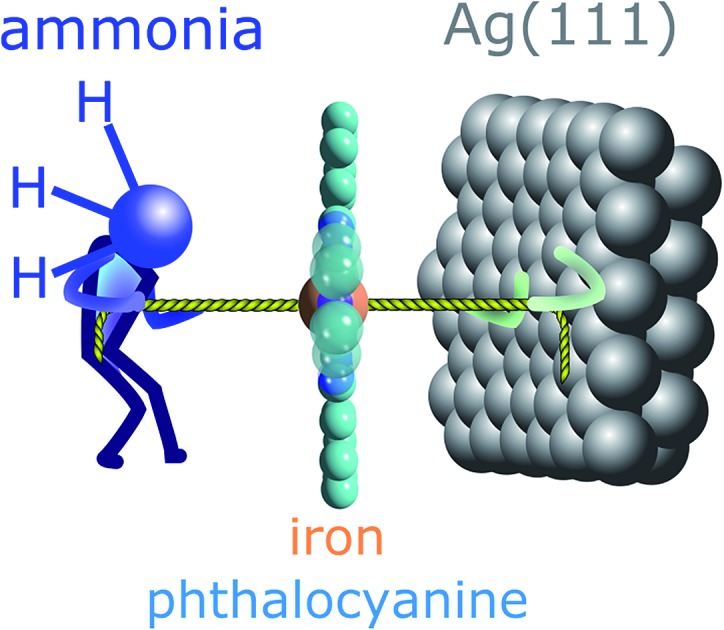
Quantification of the surface *trans*-effect: delocalised surface, rather than atomic, electrons acting as if a ligand in a traditional *trans*-effect.

## Introduction

The wealth of research of the last four decades into the adsorption of molecules and molecular ligands on metal surfaces^1^–^13^ has demonstrated the strong similarity between the behaviour of these molecule–metal interactions and those found in metal coordination compounds. In particular, the local bonding of molecules to metal substrates has been found to commonly reflect the local atomic, rather than delocalised metallic, character of the surface.^9^,^10^,^14^ Recently, spectroscopic measurements on the adsorption of planar metallo-complexes on metal surfaces^15^,^16^ have been interpreted as evidence for one further example of this analogue behaviour, namely the well-known *trans*-effect in metal coordination chemistry. Here we provide quantitative structural measurements that clearly demonstrate that this surface induced *trans*-effect does indeed occur in one such system, but, contrary to expectations of a local atomic effect, the results of complementary density functional theory (DFT) calculations show that this is a true surface effect. Specifically, we show that although the charge redistribution associated with the effect mimics that of bonding to a single atom in a coordination compound, the molecule bonds not to a single localised surface atom, but rather to the metal surface as a whole.

The study of metal–organic complexes on metal surfaces, and the way in which the substrate influences their interaction with ligand species, is motivated by the desire to design future catalysts and molecule-based devices.^15^,^17^–^30^ Of particular relevance are metal-supported porphyrins and phthalocyanines,^20^,^21^ a class of molecules with a tetrapyrrole macrocycle that can act as a chemical cavity. Into this cavity a wide variety of metal cations can be coordinated, providing these molecules with diverse functional properties for a broad range of applications in, *e.g.*, highly selective heterogeneous catalysts,^22^–^24^ molecular magnets,^25^,^26^ molecular motors,^27^ spintronics^28^,^29^ and gas sensors.^15^,^30^ However, the physical understanding and control of the processes that occur at the interface with metal supports are still in their infancy. So far, most studies conducted on these organic/metal interfaces have provided qualitative information, most prominently using scanning tunnelling microscopy (STM) (*e.g.*ref. 15, 27, 31 and 32) and X-ray photoelectron spectroscopy (XPS) (*e.g.*ref. 15, 16, 23, 24 and 32). Theoretical calculations have also been performed, but comparisons of STM images with simulated images obtained from DFT calculations do not provide unique structural solutions despite being widely exploited for this purpose. The long-standing controversy concerning the structure of the Ag(111)/p(4×4)-O phase illustrates this problem.^33^,^34^ Moreover, DFT calculations indicate that chemical shifts in the photoelectron binding energy of core level orbitals do not provide a unique indicator of adsorption sites and coordination environment.^35^ By contrast, quantitative structural measurements can provide a relatively unambiguous benchmark upon which to test theoretical predictions.^12^,^36^

One particular phenomenon that has been reported recently in this field is a significant electronic or chemical change in adsorbed metallo-porphyrin (MP) and metallo-phthalocyanine (MPc) based species after the addition of small molecular ligands to the metal centre^16^ that has been coined the surface *trans*-effect. The influence of this phenomenon has had repercussions across a wide variety of fields that utilise such supported metal–organic complexes.^25^,^37^–^40^ Of particular note is the influence it has on gas sensors: *e.g.* in the utilisation of iron phthalocyanine supported on graphene, exposure to nitric oxide apparently results in partial electron-doping of the graphene layer that manifests as a decrease in the conductance of the system.^41^ The surface *trans*-effect may also be the cause of the muted reactivity typically observed for MP and MPc on metal substrates,^24^ where the interaction between the molecule and the surface appears to prevent reaction pathways that are available to the molecule when dissolved in a solvent.

**Fig. 1:**
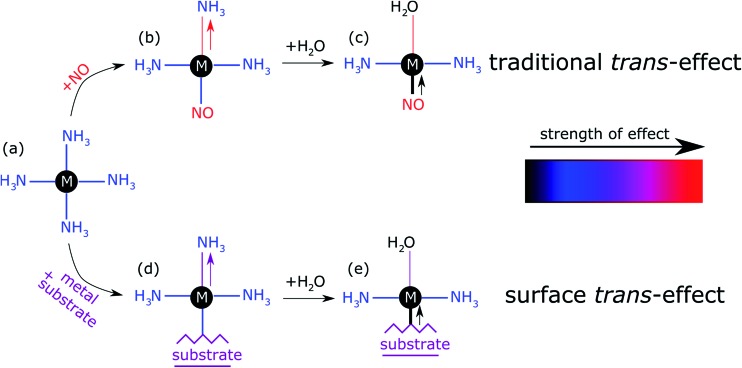
Schematic illustration of the traditional *trans*-effect and surface *trans*-effect for a hypothetical system, assuming that the intensity of the effect increases from H_2_O < NH_3_ < metal substrate < NO (indicated with the colour scheme), is shown. Specifically, a general case of (a–c) the traditional *trans*-effect where a ligand with a moderately intense *trans*-effect is replaced, sequentially, by a ligand with (a → b) a more intense and (b → c) less intense *trans*-effect; the associated change in M–ligand bond length is highlighted by the arrows. Also shown is a hypothetical case of (a → d → e) the surface *trans*-effect where a ligand with a moderately intense *trans*-effect is replaced, sequentially, by a metal substrate with (a → d) a more intense *trans*-effect and a ligand with (d → e) a less intense *trans*-effect; the associated changes in M–ligand bond length and M-substrate adsorption height (respectively) are indicated. A comparable, more realistic case of the surface *trans*-effect is shown in Fig. 2c and d for an octahedral complex.

**Fig. 2:**
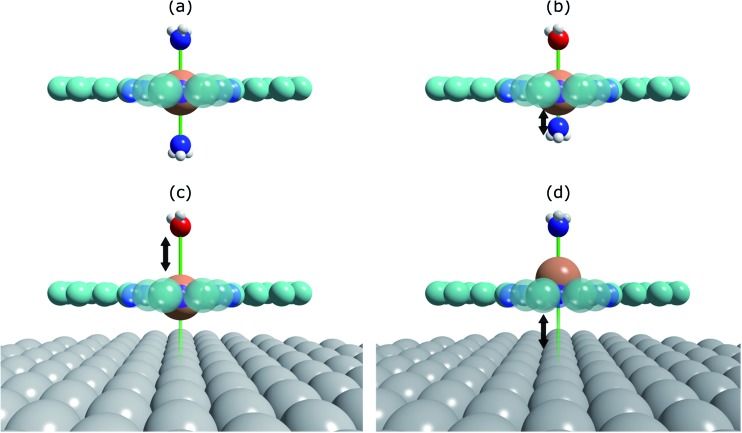
Structural schematics of (a) (NH_3_)–FePc–(NH_3_), where two ammonia molecules are *trans* to each other, (b) (H_2_O)–FePc–(NH_3_), where the ammonia molecule is *trans* to a water molecule, (c) (H_2_O)–FePc–Ag(111), where water is *trans* to the Ag(111) surface, and (d) (NH_3_)–FePc–Ag(111), where ammonia is *trans* to the Ag(111) surface. Assuming the order of the intensity of the *trans* effect goes: H_2_O < NH_3_ < Ag(111), then the traditional *trans*-effect would suggest a shorter Fe–(NH_3_) bond when ammonia is *trans* to a water molecule, than when it is *trans* to another ammonia molecule. Similarly, a longer Fe–(H_2_O) bond would be expected when water is *trans* to the Ag(111) surface than to ammonia. Finally, this would also imply a larger adsorption height of the Fe centre when the Ag(111) surface is *trans* to ammonia than to water.

The prototypical system, when MP and MPc molecules are adsorbed on a metallic substrate, has the central molecular macrocycle orientated (approximately) parallel to the substrate plane.^42^,^43^ It is this adsorption geometry that leaves the centrally coordinated metal ion close to the substrate, but also free to interact with potential ligand molecules at the position *trans* to (*i.e.* opposite to) the substrate. Flechtner *et al.*^15^ reported that when cobalt tetraphenyl porphyrin (Co-TPP) was adsorbed onto Ag(111), a significant difference in Co 2p photoelectron binding energy was observed in XPS between single-layer and multilayer samples. However, this energy difference was greatly reduced following exposure to NO. This experimental finding was tentatively ascribed to a weakening of the interaction between the metal centre of the porphyrin and the substrate caused by the ligation of NO at the position *trans* to the substrate. This was interpreted as being, at least phenomenologically, similar to the traditional *trans*-effect observed in coordination chemistry,^44^–^46^ in which a ligand with an intense *trans*-effect, either through being a strong σ-donor or π-acceptor, weakens the ligand–metal bond that is *trans* to it. In Fig. 1 a phenomenon associated with the traditional *trans*-effect is illustrated by a hypothetical scheme. Here, a ligand with an intense *trans*-effect, NO, replaces an NH_3_ molecule (Fig. 1a and b), weakening the NH_3_–metal bond that is *trans*/opposite to it, and thus promoting the replacement of this ammonia molecule by a second ligand (Fig. 1b and c). A consequence of this is that an intense *trans*-effect ligand induces a longer metal–ligand bond length at the *trans* position (Fig. 1b), whereas a milder *trans*-effect ligand results in a shorter metal–ligand bond at the trans position (Fig. 1c). Similarly, when dealing with octahedral coordination, as in the case of molecular ligation to phthalocyanines and porphyrins, if molecular ammonia is replaced by molecular water, a ligand with an even milder *trans*-effect than ammonia, this replacement will lead to a shortening of the bond length of the remaining ammonia molecule that is *trans* to it (Fig. 2a and b). In the proposed surface *trans*-effect, the surface plays the role of one of the ligands, not only *inducing* a *trans*- (or *trans*-like-) effect, as shown in Fig. 1d and 2c, but also *experiencing* a *trans*- (or *trans*-like-) effect, as shown in Fig. 1e and 2d. This interpretation was tested by Hieringer *et al.*,^16^ who probed the interaction of Fe-TPP, Co-TPP and Zn-TPP with NO on the Ag(111) surface using ultraviolet photoelectron spectroscopy, XPS and STM, and qualitatively comparing the shifts in the Co 2p XPS binding energy with those obtained from DFT calculations. These DFT calculations predicted a significant structural displacement of the metal ion by >0.6/0.7 Å for Co and >0.4/0.7 Å for Fe (PBE/PBE + vdW). Since the original work of Flechtner *et al.* several other groups have observed various electronic and chemical effects that have all been attributed to this “surface *trans*-effect”^21^,^24^,^25^,^37^–^40^,^47^. However, prior to our study, there have been no investigations of the predicted structural changes, nor has there been a quantitative comparison of theoretical predictions to experimental results.

**Fig. 3:**
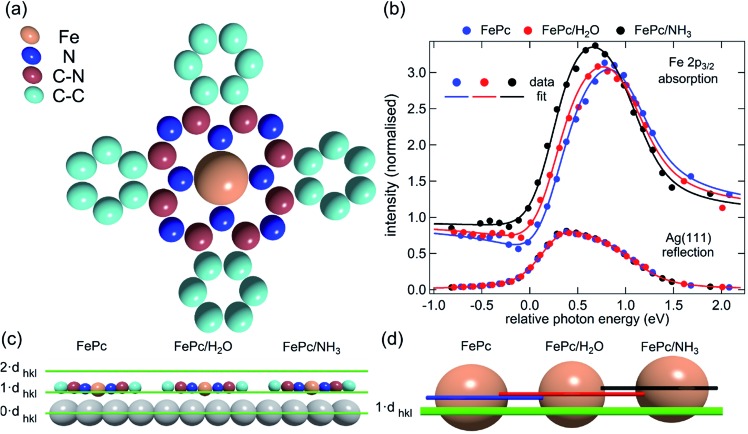
(a) Molecular structure of FePc. Note that the two different species of C, which could be chemically resolved in the XSW analysis as shown in Fig. S1a,† are assigned to C atoms bound to C and N atoms (C–N) and bound only to other C atoms (C–C). (b) Comparison of the Fe 2p_3/2_ X-ray absorption profiles before molecular ligation (FePc), after water ligation (FePc/H_2_O) and after ammonia ligation (FePc/NH_3_). (c) A to scale schematic representation of the quantitative analysis of the XSW data detailed in Table 1. The atomic scattering plane at the surface termination (0·*d*_*hkl*_), the first (1·*d*_*hkl*_) and the second (2·*d*_*hkl*_) extended planes above the surface are also indicated. Also shown (d) is a 4× magnification of the displacement of the Fe atoms with horizontal lines indicating the centre of the Fe atoms above the first extended surface plane (1·*d*_*hkl*_).

Here we present the results of such a quantitative experimental test of DFT predictions, utilising normal incidence X-ray standing waves (NIXSW)^48^ to measure the displacement of iron phthalocyanine (FePc – shown schematically in Fig. 3a), adsorbed on a single crystal Ag(111) surface, before and after ligation of ammonia and water. The choice of system was inspired by the results of published DFT calculations (which did not include dispersion forces) predicting a 0.9 Å displacement of the iron centres of FePc on the Au(111) surface upon ligation of ammonia, as well as by a report of experimentally observed electronic changes indicative of the “surface *trans*-effect”^49^.

The NIXSW^48^ technique exploits the standing wavefield generated by the interference between an incident photon beam and its reflected component at a Bragg condition. As the incident photon energy is scanned through the Darwin reflectivity curve, the antinodes of the standing wave move from being half way between the atomic scattering planes to being coincident with them. As the standing wave extends beyond the termination (surface) of the substrate, adsorbates that lie above the surface will experience varying electromagnetic field intensity as a function of the incident photon energy, dependent on their position relative to the extended scatterer planes. Therefore, monitoring the X-ray absorption at an adsorbate atom as a function of photon energy yields a profile indicative of the average position (coherent position) of the atom relative to the scattering planes of the substrate, and a parameter related to the fraction of atoms that occupy that position (coherent fraction). By using normal incidence to scatterer planes that are parallel to the surface, the coherent position for an adsorbate species becomes equal to the average height of the adsorbate atom above the outermost substrate layer, provided that the relaxation of the outermost substrate layers can be neglected.

## Methods

### Experimental section

The XSW measurements were performed at the I09 beam line at the Diamond Light Source. The intensity and width of the (111) Bragg reflection of Ag, measured at almost exactly normal incidence, (∼2640 eV at ∼60 K) was acquired from a fluorescent screen mounted on the port through which the incident photons passed. The Darwin reflectivity curve was used to define the energy scale with respect to the Bragg energy, the energy broadening due to imperfections in the monochromator (Si(111) double-crystal) and the mosaicity of the single crystal substrate (which was found to be negligible). The experimental chamber was orientated with either a 60° or 90° angle between the incident photon beam and the centre of the detector, a VG Scienta EW4000 HAXPES hemispherical electron analyser with an angular acceptance range of ±30°. The integrated intensities of the Fe 2p, N 1s and C 1s photoemission peaks were used to monitor the relative X-ray absorption of the Fe, N and C atoms, respectively. Both instrumental geometries led to similar values for the NIXSW fitting parameters once non-dipolar effects in the angular dependence of the photoemission were taken into account. Specifically, the backward-forward asymmetry parameter *Q* was calculated theoretically using the average angle acquired on the analyser (*θ* = 30° and *θ* = 18° respectively, as defined in ref. 50). To minimise radiation damage to the adsorbed molecules the sample was held at ∼60 K, and the X-ray beam (defocused to approximately 300 × 300 μm^2^) was stepped over the sample during each XSW measurement such that each energy point in a single scan was acquired from a different position on the sample. The base pressure in the end station was ∼5 × 10^–10^ mbar, which necessitated re-preparation of the sample every ∼8 hours to limit the adsorption of residual water in the vacuum. A clean Ag(111) crystal was prepared by repeated cycles of Ar^+^ sputtering and annealing to 800 K for 25 minutes. A multilayer of FePc was deposited by sublimation of FePc powder (Sigma Aldrich, 90% pure by dye content, triply purified^51^) at 680 K onto the sample held at room temperature. The sample was then annealed to ∼600 K for 40 minutes, desorbing the excess FePc and resulting in a low energy electron diffraction pattern consistent with a saturated incommensurate single-layer described by a matrix of 
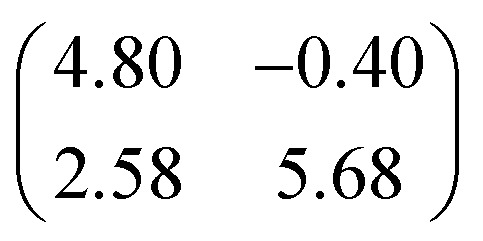
.^52^ This Ag(111)/FePc system was subsequently exposed to ammonia or water by backfilling of the chamber to pressures of 10^–8^ mbar with the substrate held at 60 K. Due to the placement of the ion gauge near the turbomolecular pump, at a significant distance from the sample, an accurate measure of the exposure rate was not possible; however the coverage of all exposures was monitored by XPS measurements (not shown) and indicated in all cases a coverage of ammonia or water greater than around 1 molecule per 3 silver surface atoms (∼0.33 ML), which is greater than that required to saturate each Fe atom (∼0.04 ML). Between ∼0.33 ML and ∼3 ML, no trend was observed in the variation of the height of the FePc molecules, as a function of ammonia (or water) coverage. At each stage of the preparation XPS was utilised to monitor any possible contaminants, and, with the exception of the aforementioned adsorption of residual water in the vacuum and damage caused by the X-ray beam after lengthy exposure, none were observed. An in-depth discussion of the data reduction will be reported in a later publication, but in all cases a Voigt lineshape was used to model the photoemission peaks, except the Fe 2p_3/2_ multiplet structure, which was modelled with a Gaussian lineshape. The backgrounds of the Fe 2p_3/2_ spectra were fitted using a template background (measured over the same energy range on the clean Ag(111) substrate), whereas Shirley backgrounds were used for the N 1s and C 1s spectra. For the N 1s spectra two Gaussians were also applied to compensate for the underlying Ag plasmons.

### Computational section

The density functional calculations were carried out using the VASP package^53^. The PBE–GGA exchange–correlation potential^54^ was used, and the electron-core interactions were treated in the projector augmented wave method.^55^,^56^ The van der Waals interaction has been taken into account through the so-called DFT-D2 and D3 semi-empirical methods *via* a pair-wise force field^57^,^58^ or by using a non-local optB88-vdW (DFT-vdW) exchange–correlation functional.^59^,^60^ All the calculations were performed using a four-layer Ag slab embedded in 15 Å of vacuum space, ensuring 10 Å separation between adjacent supercells. Structures were optimised using energies calculated at a single *k* point (the *Γ* point of the Brillouin zone) with a kinetic cut-off energy of 400 eV. The coordinates of the uppermost Ag layer and the molecules were fully relaxed until the atomic forces were smaller than 0.01 eV Å^–1^. When the DFT-D2 method was used, the van der Waals interaction of either only the top layer (DFT-D2-1L), or all the layers (DFT-D2), was taken into account in the pairwise interaction^57^ (DFT-D2-1L results are not presented below, but can be found in the ESI, Tables S2–S4†).

## Results

The XSW absorption profiles, monitored by the Fe 2p_3/2_ photoemission, from a single-layer of adsorbed FePc, both before and after exposure to ammonia or water, show a clear shift of the maximum of the profile to lower photon energies following molecular uptake (Fig. 3b). This shift indicates that there is an increase in the coherent position, and thus in the height of the adsorbed Fe atom above the Ag(111) surface, due to additional molecular ligation. Specifically, the results shown in Fig. 3b indicate that the Fe atom is at its lowest position prior to ligation, at its highest position when ligated to ammonia, and at an intermediate position when ligated to water. Quantitative analysis of the absorption profiles show that the average height of the Fe atom changes by +0.19 ± 0.07 Å after the adsorption of ammonia, and by +0.07 ± 0.04 Å after the adsorption of water. Similar effects, though significantly smaller, were observed for the absorption profiles recorded for the carbon and nitrogen atoms, as shown schematically in Fig. 3c and detailed in Table 1 (coherent positions, coherent fractions and absolute heights shown in the ESI, Table S1†), indicating that there is a shift of the whole molecule to greater heights above the surface.

**Table 1:** Comparison of the experimental and theoretical displacements in the FePc height, in ångström units, above the surface (averaged over the molecule in an atop, bridge and hollow site) for the Fe, C and N atoms upon ligation of NH_3_ (Ag(111)/FePc/(NH_3_)–Ag(111)/FePc) and H_2_O (Ag(111)/FePc/(H_2_O)–Ag(111)/FePc). The number in brackets is the uncertainty (standard error at two standard deviations) in the last decimal place. The absolute heights from XSW and DFT analysis can be found in the ESI

	Ag(111)/FePc/(NH_3_)–Ag(111)/FePc				Ag(111)/FePc/(H_2_O)–Ag(111)/FePc			
	Fe	N	C–N	C–C	Fe	N	C–N	C–C
XSW (Å)	0.19(7)	0.13(7)	0.07(6)	0.06(6)	0.07(4)	0.08(9)	0.01(2)	–0.02(5)
DFT (Å)	1.10	0.82	0.78	0.51	0.15	0.06	0.05	0.01
DFT-D2 (Å)	0.19	0.09	0.08	0.03	0.07	0.03	0.03	0.01
DFT-vdW (Å)	0.22	0.12	0.11	0.06	0.04	0.02	0.02	0.02
DFT + D3 (Å)	0.31	0.16	0.14	0.06	0.07	0.02	0.01	0.00

Also reported in Table 1 are the atomic displacements predicted by DFT calculations. The results from the DFT-D2 calculations agree surprisingly well with the experimental XSW results. The DFT-D3 calculations significantly overestimated the effect of ammonia ligation, most notably to the Fe metal centre. The DFT-vdW calculations using optimised exchange energy also show results comparable to the experiments, but with better agreement for the absolute positions, which were overestimated for both DFT-D2 and DFT-D3 (see ESI, Tables S2–S4†). It should be noted that due to the large size of the system, hybrid functional and random phase approximation (RPA) calculations are impractical, though it is anticipated that the correction of the self-interaction using the hybrid functional will not significantly affect the predicted atomic displacement that is the focus of the present investigation.

In contrast, theoretical calculations without van der Waals corrections predict a significantly larger displacement of the entire molecule than is experimentally observed. However, the qualitative trends, *e.g.* a smaller displacement due to water adsorption and a smaller displacement of the molecular backbone than of the Fe atom, are reproduced. Inclusion of van der Waals corrections into the calculations decreases the size of the displacement dramatically for both ligands, bringing the theory into excellent quantitative agreement with the experimental measurements.

## Discussion

The observed displacement of the Fe centre of FePc is qualitatively consistent with the results of the DFT calculations of Hieringer *et al.*;^16^ the weakening of the metal centre interaction with the metal substrate does indeed have a structural effect similar to that expected from the traditional *trans*-effect in coordination chemistry. It has long been established that adsorbates on metal surfaces follow rules comparable to those developed in coordination chemistry,^9^,^10^,^61^ so extending this analogous behaviour to metal–organic complexes adsorbed on metal surfaces may appear entirely reasonable. However, one should question the “surface *trans*-effect” terminology, because the comparison with metal coordination compounds effectively requires the surface to be considered not only as having an effect comparable to a ligand, but one whose interaction with the metal centre of the phthalocyanine has a specific directionality. In other words, to be physically a *trans*-effect the interaction cannot be mediated through long-range forces (*e.g.* induced dipoles or vdW forces), instead it must be induced through the sharing of an orbital of the metal centre by both the surface and the ligand *trans* to it, in order to satisfy either a σ-donor or a π-acceptor interpretation of the traditional *trans*-effect.^44^,^45^ The hybridisation of metal adatoms to metal surface atoms, when coadsorbed with molecules to form metal–organic coordination networks, has been proposed from theoretical calculations.^62^,^63^ However, it is not clear to what extent this is comparable to the interaction between the metal surface and the metal centres within adsorbed MP and MPc molecules. This raises the question: if it looks like the *trans*-effect, acts like the *trans*-effect, does it actually mean it is the *trans*-effect? In other words, is the term “surface *trans*-effect” merely a useful description of the observed phenomena in broad terms, or does it have actual physical meaning?

The overlayer studied here, a saturated single-layer of FePc on Ag(111), is known to be incommensurate with the underlying substrate.^52^ However, the coherent fractions observed for the adsorbed molecule are relatively high (see Table S1†), so the variation in the height of the molecules above the surface is small, despite the local coordination of the Fe atom varying over an effective infinity of sites. If the Fe-substrate height were exactly the same throughout the incommensurate overlayer then the local Fe–Ag atomic distance would increase as the number of nearest neighbour Ag atoms increases (*e.g.* the distance is longer over a hollow site than over an atop site). This is qualitatively similar to what is observed for small molecular species when adsorbed in atop, bridge and hollow sites^9^ on metal surfaces, an effect interpreted as following rules similar to those devised for coordination chemistry. However, this does not necessarily suggest that, if the interaction between the metal complex and the metal substrate follows the same general trend, then the Fe_ads_–Ag_surf_ interaction would also be comparable to that of the ligand to metal centre interaction in coordination chemistry. More generally, as there are effectively an infinite number of different local adsorption sites in the incommensurate layer, it is clear that most Fe atoms will not be directly above a Ag atom, so there will not be a silver atom in a site *trans* to the adsorbed molecular ligand; in contrast, the traditional *trans*-effect clearly suggests a strong directional influence. This implies that if the “surface-*trans*-effect” is indeed a *trans*-effect, then the substrate to metal complex interaction cannot be mediated by direct interaction to the silver atoms, instead it must be the delocalised metal surface electrons that drive the effect.

**Fig. 4:**
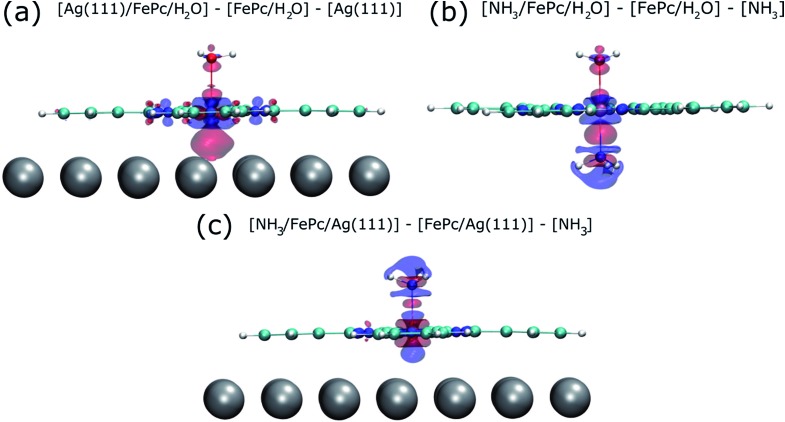
Charge redistribution map (CRM) showing an isosurface plot with ±0.02 e Å^–3^ (red is an increase, blue a decrease in charge density). Shown is (a) the difference caused by adding the Ag(111) surface *trans* to a water molecule, (b) by adding ammonia *trans* to a water molecule and (c) by adding ammonia *trans* to the Ag(111) surface. More details are given in the ESI (Fig. S2†).

**Fig. 5:**
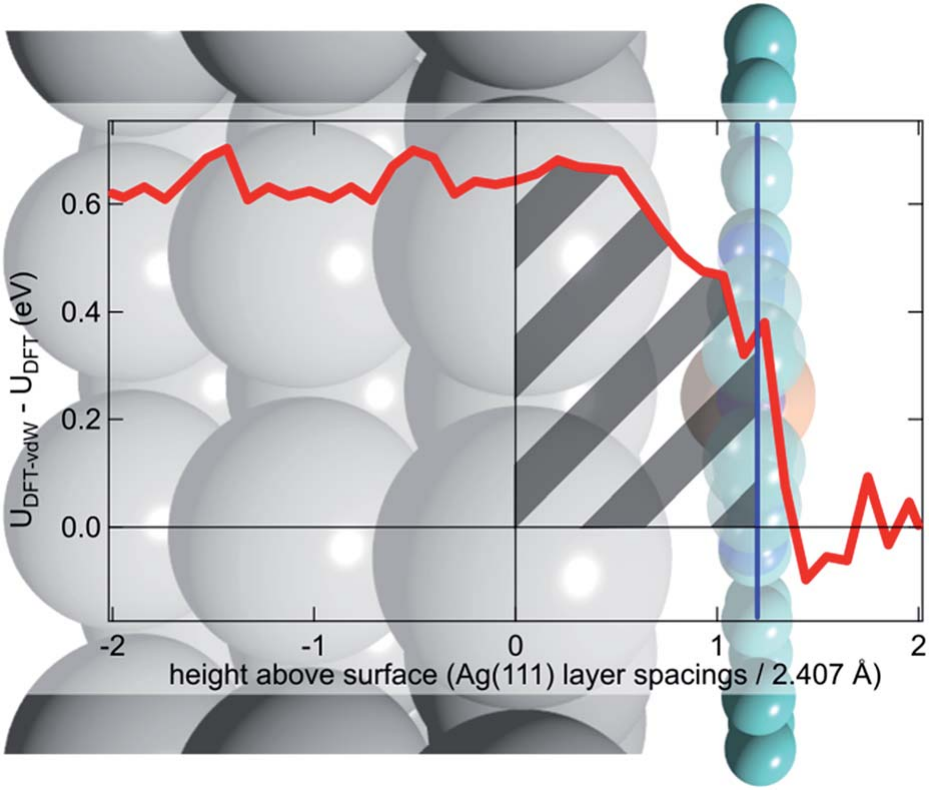
Difference between the optB88-vdW and PBE functionals in the calculated plane-averaged electrostatic potential from a clean Ag surface (red line), the termination of which is set at 0, overlaid atop a schematic of Ag(111)/FePc. The adsorption height of the FePc is also indicated (blue line) showing that at the position occupied by the FePc molecule the dispersion corrected DFT calculations predict a more positive potential. We posit that this allows a greater accumulation of charge between the surface and the adsorbed FePc (shaded grey), intensifying the *trans*-effect of the surface. Further details are given in the ESI.†

To try to address these issues, calculations for a number of simple model structures have been conducted, encouraged by the good quantitative agreement between the theory and the experiment in reproducing the structural consequences of this “surface *trans*-effect”. Specifically, further calculations were performed on traditional *trans*-effect systems, comparing (NH_3_)–FePc–(NH_3_), (H_2_O)–FePc–(H_2_O), (NH_3_)–FePc–(H_2_O) and (NH_3_)–FePc–(NO). The N–Fe and O–Fe bond lengths are listed in the ESI (Table S5†) and compared to those of the relevant Ag(111)/FePc calculations; these values show that the effect of the Ag substrate is to induce a bond length between the *trans*-ligand and the Fe atom that is significantly longer than that induced by ammonia or water, but shorter than that induced by NO. This difference in bond length would place the silver surface as having a potential *trans*-effect somewhere between the weak (water, ammonia) and the strong (NO) ligands. When charge redistribution maps (CRMs) are compared (Fig. 4) it can be seen that the effects on the Fe centre of introducing the Ag surface (Fig. 4a) or an NH_3_ ligand (Fig. 4b) *trans* to a water molecule are remarkably similar. In both cases there is significant redistribution of charge into the σ-bonds that lie between the introduced species (Ag(111)/NH_3_) and the Fe centre, while a smaller increase in charge density occurs between the O and Fe atoms. This is mostly manifest as an accumulation of charge density in the Fe d_*z*^2^_ orbital and a comparable decrease in the Fe d_*xz*_ and d_*yz*_ orbitals. Introducing an NH_3_ ligand (Fig. 4c) *trans* to the Ag(111) substrate has an effect on the NH_3_ species similar to that produced when it is introduced *trans* to a water ligand (Fig. 4b). However, Fig. 4c shows the opposite effect occurs at the Fe metal centre (charge depletion in d_*z*^2^_ and accumulation in d_*xz*_ and d_*yz*_) when compared to the behaviour seen in both Fig. 4a and b. This might result from the Ag(111) having a stronger *trans*-effect than the NH_3_ ligand, indicated by the longer *trans*-ligand to Fe bond length for Ag, shown in Table S5.† The CRMs including Ag(111) shown in Fig. 4 and S2† are calculated for the Fe centre above a hollow site, but similar calculations (not shown here) for an atop and a bridge site show the same effect, further indicating that it is the interaction with the delocalised metal electrons of the substrate, rather than a direct interaction with a substrate atom that drives the surface *trans*-effect. These calculations thus indicate that the same effect would be seen for both commensurate and incommensurate overlayers. The intensity of the surface *trans*-effect seen on Ag(111) appears to be dependent upon the ability to accumulate charge between the Ag(111) surface and the FePc. This may explain why the inclusion of dispersion forces dramatically changes the predicted structural differences induced by the surface *trans*-effect. The vdW corrections predict a less negative electrostatic potential for the clean Ag(111) surface than the uncorrected calculations do (Fig. 5). Specifically at the measured adsorption height of the FePc molecule (∼2.8 Å, corresponding to 1.2 Ag substrate layer spacings) the potential, with respect to the vacuum level, is less negative by ∼0.4 eV. Therefore, in the region between the surface and the adsorbed molecule there is a significant decrease in the energy required to remove an electron to the vacuum level, *i.e.* the local work function has decreased. We posit that this lowering of the local work function, when using the vdW-DF functional, allows a greater charge accumulation in the σ-bonding area intensifying the Ag(111) surface *trans*-effect. Such a sensitivity to change in the local work function would reinforce the idea that the surface *trans*-effect arises from interactions with the electrostatic potential of the substrate, rather than being mediated through direct interaction with any individual atom. The theoretical calculations thus predict that the “surface *trans*-effect” is mediated through redistribution of charge along the *trans* σ-bonds in a manner similar to that of the traditional *trans*-effect.

## Conclusions

Utilising the X-ray standing wave technique, we have obtained the first quantitative structural measurement of the consequences of the “surface *trans*-effect”, observing the displacement of the metal centre of a metal–organic species by its ligation to a molecular ligand. This displacement has been modelled by DFT calculations that show excellent agreement with the experimental results, provided that corrections are included for dispersion forces. Furthermore, these dispersion-corrected calculations predict that the electronic effect on the FePc/water moiety of introducing the Ag(111) surface is remarkably similar to that of introducing an ammonia molecule. However, the main difference between the traditional *trans*-effect and the surface *trans*-effect would seem to be that, rather than involving a direct interaction between the metal complex centre and an atom in the coordinating ligand/surface, it is the interaction between the metal complex centre and the delocalised electronic states of the metal substrate that drives the surface *trans*-effect.

This result has wide-ranging implications in the field of metal–organic complexes supported on metal substrates. The most obvious relevance is for potential catalysts, especially considering adsorbed planar species like MPs and MPcs, as the active site is inherently *trans* to the substrate, and the coordination of a ligand *trans* to the substrate gives rise to the activated complex of such a catalyst. It can be inferred that the weakening of the metal centre–ligand interaction by the substrate will suppress the reactivity of the adsorbed complex, suggesting that one cannot simply adsorb a liquid or gas phase catalyst onto a metal substrate and expect comparable activity. Instead, as concluded in our previous published work^24^ it may be necessary to choose metal centres that are traditionally seen as being “too reactive” for catalytic reactions. It is also not unreasonable, as was proposed by Hieringer *et al.*,^16^ to expect that different substrates will have varying intensities of the surface *trans*-effect. This extra degree of freedom could be exploited to tune the selectivity of such a catalyst, for example, in electrochemical oxygen^32^ and carbon dioxide^64^ reduction, where the metal–organic complexes are positioned on a metallic electrode. In a similar manner, the consequences for the design of electronic devices that assume the substrate to be an inert component will be deleterious; the influence of the substrate must be considered from the outset. On the other hand, this result is potentially promising in the field of adsorbed gas sensors. As the surface *trans*-effect not only affects the ligand to metal centre interaction, but also the surface to metal centre interaction, the response of the adsorbed metal complex to a gaseous species will induce an effect on the substrate, potentially amplifying the response of the gas sensor as was observed for NO ligation to FePc on graphene.^41^

As it has now been clearly demonstrated that the surface *trans*-effect occurs in a manner similar to that in traditional coordination chemistry, it raises the question as to whether there is a comparable *cis*-effect. The *cis*-effect is considerably less studied in coordination chemistry than the *trans*-effect, but can have a similar influence on the reactivity of a metal complex, especially in octahedral systems. However the experimental results presented here provide no evidence for such an effect, though some redistribution of charge in the plane of the FePc molecule was seen in the CRM's (see Fig. 4a). It is therefore probable that a surface *cis*-effect does indeed exist, hence a study like that presented here, but investigating a system where a potential *cis*-effect would be expected to dominate, could be fruitful.

Finally, the prediction of the remarkable similarity of the electronic effect of the “surface *trans*-effect” to the traditional *trans*-effect, as shown in the charge redistribution maps, strongly suggests that this phenomenon is truly a *trans*-effect, not only in appearance, but also in physical manifestation. Surprisingly, though it manifests as a *trans*-effect, this effect is not due to an interaction with a single substrate atom, but instead with the surface delocalised electrons making this a true surface, rather than local, effect, thus the name “surface *trans*-effect” is fully justified.

## Supplementary Material

Supplementary information
