# Supplementary material for: Direct quantitative identification of the “surface trans-effect”
Source: Chem Sci. 2016 Jun 9;7(9):5647–56. doi: 10.1039/c6sc01677d (PMC6022009; doi:10.1039/c6sc01677d)
Supplement: Supplementary file 1 [file SC-007-C6SC01677D-s001.pdf]

Electronic Supplementary Material (ESI) for Chemical Science.
This journal is © The Royal Society of Chemistry 2016

Direct quantitative identification of the "surface *trans*-effect"

Peter S. Deimel[a], Mohammed R. Bababrik[b], Bin Wang[b], Phil J. Blowey[c,d], Luke A. Rochford[e], Pardeep K. Thakur[c], Tien-Lin Lee[c], Marie–Laure Bocquet[f], Johannes V. Barth[a], D. Phil Woodruff[d], David A. Duncan[a,c]* and Francesco Allegretti[a]*

[a]Physics Department E20, Technical University of Munich, 85748 Garching, Germany

[b]Center for Interfacial Reaction Engineering and School of Chemical, Biological and Materials Engineering, the University of Oklahoma, Norman, 73019-1004 Oklahoma, USA

[c]Diamond Light Source, Harwell Science and Innovation Campus, Didcot, OX11 0QX UK

[d]Department of Physics, University of Warwick, Coventry, CV4 7AL, UK

[e]Department of Chemistry, University of Warwick, Coventry, CV4 7AL, UK

[f]ENS – Department of Chemistry, PSL Research University, CNRS UMR 8640 PASTEUR, 75005 Paris, France

*DA.Duncan@tum.de and Francesco.Allegretti@ph.tum.de

XPS

X-ray photoelectron spectra of the iron phthalocyanine (FePc) saturated single layer before exposure to ammonia or water acquired using the soft X-ray light available at the I09 beam line are shown in FIG. S1. Both the Fe $2p_{3/2}$ and N 1s core levels partially overlap with bulk spectral features, specifically the tail of the Ag 3s and the vibrational loss features of the Ag 3d core levels (respectively). The Fe $2p_{3/2}$ line is split into two components (Fig. S1b), a main peak and a broader energy loss feature attributed to the unpaired electrons in the d-orbitals[S1]. The C 1s line has two main components related to the C atoms bonded to N atoms (C-N, Fig. S1a,d) and C atoms bonded to only C atoms (C-C, Fig. S1a,d). The N 1s spectrum contains only the single peak relating to all 8 N atoms in the phthalocyanine molecule (Fig. S1c,d).

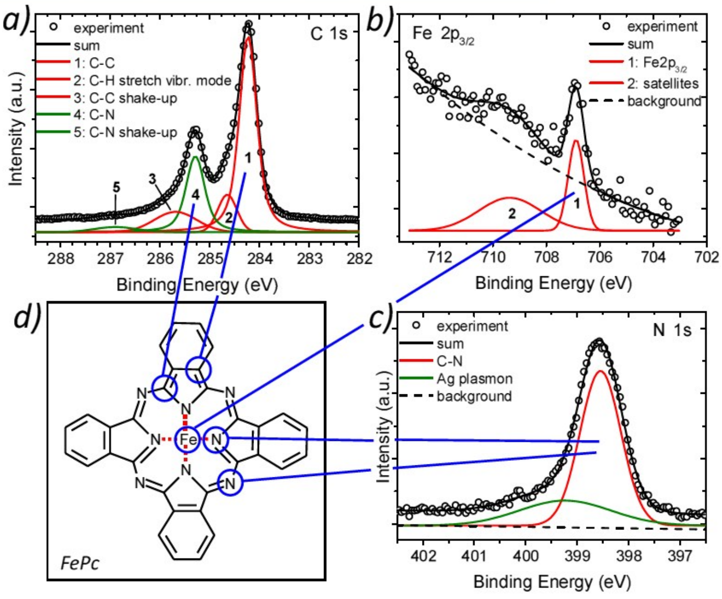

Figure. S1: (a) C 1s (hν = 435 eV), (b) Fe 2p (hν = 880 eV) and (c) N 1s (hν = 550 eV) XP spectra measured from a FePc monolayer on Ag(111). The assignment of the C 1s spectra follows that proposed by N. Parageorgiou et al.[S2]. Also shown (d) is a molecular schematic of the FePc molecule and the assignment of the species observed in the XP spectra.

Structural parameters

The absolute heights for the FePc molecule before and after the ligation of ammonia and water are shown in Table S1, along with the corresponding coherent fractions and coherent positions. The comparable absolute heights from the DFT calculations using the functionals described above, are also reported for the center of the molecule above a hollow site, atop site and bridge site for iron phthalocyanine adsorbed on Ag(111) without any ligand (Table S2), with ligated ammonia (Table S3) and with ligated water (Table S4). Shown in Table S5 are the metal-ligand distances for chosen traditional *trans*-effect and surface *trans*-effect systems.

Table S1: Coherent fractions (F_c) and coherent positions (P_c) found experimentally in this study for Fe, N and C atoms (C split into C-C and C-N species as identified in FIG. S1), as well as the height of the various species above bulk like termination of the Ag(111) surface. Note D(hkl) for Ag(111) is taken as 2.35 Å, and it is assumed that the molecule is over one layer spacing above the surface (otherwise unphysical results are obtained).

Ag(111)/FePc	Fe	N	C-N	C-C
F_C	0.9(1)	0.87(5)	0.9(1)	0.9(2)
P_C	0.11(1)	0.15(3)	0.20(2)	0.24(2)
D(hkl)+D(hkl)*P_H (Å)	2.61(1)	2.71(7)	2.83(5)	2.92(5)

Ag(111)/FePc/NH_3				
F_C	0.73(8)	0.9(3)	0.9(1)	0.95(11)
P_C	0.19(3)	0.21(1)	0.23(1)	0.27(1)
D(hkl)+D(hkl)*P_H (Å)	2.80(7)	2.84(2)	2.90(3)	2.98(3)

Ag(111)/FePc/H_2O				
F_C	0.75(7)	0.8(1)	0.9(1)	0.9(1)
P_C	0.14(2)	0.19(3)	0.21(1)	0.23(4)
D(hkl)+D(hkl)*P_H (Å)	2.68(4)	2.79(6)	2.84(2)	2.90(1)

Table S2: Absolute heights of the various components of the molecule above the Ag(111) surface as calculated for Ag(111)/FePc by DFT calculations with different methods to include the van der Waals interaction. The weighted mean is assuming that in each surface unit mesh there is one unique atop site, two unique bridge sites and two unique hollow sites (assuming the FCC hollow site is identical to the HCP hollow site). When calculating the height of the atoms, the average height of the First-Layer Ag atoms has been used as the reference.

		Fe (Å)	N (Å)	C-N (Å)	C-C (Å)
DFT+D2-1L	Top	2.80	2.87	2.88	2.86
	HCP	2.72	2.84	2.86	2.86
	Bridge	2.71	2.83	2.85	2.86
	mean	2.74	2.85	2.86	2.86
	weighted	2.73	2.84	2.86	2.86
	std. dev.	0.05	0.02	0.02	0.00
DFT+D2-4L	Top	2.77	2.83	2.83	2.80
	HCP	2.65	2.77	2.79	2.81
	Bridge	2.63	2.76	2.78	2.80
	mean	2.68	2.79	2.80	2.80
	weighted	2.67	2.78	2.79	2.80
	std. dev.	0.08	0.04	0.03	0.01
DFT+D3	Top	2.82	2.95	2.97	3.02
	HCP	2.73	2.90	2.93	3.00
	Bridge	2.69	2.87	2.90	3.01
	mean	2.75	2.91	2.93	3.01
	weighted	2.73	2.90	2.93	3.01
	std. dev.	0.07	0.04	0.04	0.01
DFT+ optB88-VdW	Top	2.80	2.87	2.88	2.88
	HCP	2.60	2.77	2.79	2.89
	Bridge	2.59	2.74	2.77	2.90
	mean	2.66	2.79	2.81	2.89
	weighted	2.64	2.78	2.80	2.89
	std. dev.	0.12	0.07	0.06	0.01

Table S3: Absolute heights of the various components of the molecule above the Ag(111) surface as calculated for Ag(111)/FePc/NH_3 by DFT calculations with different methods to include the van der Waals interaction. The weighted mean is assuming that in each surface unit mesh there is one unique atop site, two unique bridge sites and two unique hollow sites (assuming the FCC hollow site is identical to the HCP hollow site). When calculating the height of the atoms, the average height of the first-Layer Ag atoms has been used as the reference.

		Fe (Å)	N (Å)	C-N (Å)	C-C (Å)
DFT+D2-1L	Top	3.00	2.98	2.97	2.90
	HCP	2.90	2.92	2.93	2.89
	Bridge	2.91	2.92	2.92	2.89
	mean	2.94	2.94	2.94	2.89
	weighted	2.93	2.93	2.94	2.89
	std. dev.	0.08	0.03	0.03	0.01
DFT+D2-4L	Top	2.96	2.93	2.92	2.84
	HCP	2.84	2.86	2.86	2.83
	Bridge	2.83	2.84	2.85	2.82
	mean	2.88	2.88	2.88	2.83
	weighted	2.86	2.87	2.87	2.83
	std. dev.	0.07	0.05	0.04	0.01
DFT+D3	Top	3.14	3.11	3.11	3.08
	HCP	3.01	3.04	3.05	3.05
	Bridge	3.02	3.04	3.05	3.08
	mean	3.06	3.06	3.07	3.07
	weighted	3.04	3.05	3.06	3.07
	std. dev.	0.07	0.04	0.03	0.02
DFT+ optB88-VdW	Top	3.00	2.98	2.98	2.94
	HCP	2.85	2.89	2.91	2.96
	Bridge	2.81	2.86	2.88	2.96
	mean	2.89	2.91	2.92	2.95
	weighted	2.86	2.90	2.91	2.96
	std. dev.	0.10	0.06	0.05	0.01

Table S4: Absolute heights of the various components of the molecule above the Ag(111) surface as calculated for Ag(111)/FePc/H_2O by DFT calculations with different methods to include the van der Waals interaction. The weighted mean is assuming that in each surface unit mesh there is one unique atop site, two unique bridge sites and two unique hollow sites (assuming the FCC hollow site is identical to the HCP hollow site). When calculating the height of the atoms, the average height of the first-Layer Ag atoms has been used as the reference.

		Fe (Å)	N (Å)	C-N (Å)	C-C (Å)
DFT+D2-1L	Top	2.87	2.90	2.91	2.87
	HCP	2.78	2.87	2.88	2.87
	Bridge	2.78	2.86	2.87	2.88
	mean	2.81	2.88	2.89	2.87
	weighted	2.80	2.87	2.89	2.87
	std. dev.	0.05	0.03	0.04	0.01
DFT+D2-4L	Top	2.84	2.86	2.86	2.81
	HCP	2.72	2.81	2.82	2.81
	Bridge	2.71	2.79	2.80	2.81
	mean	2.76	2.82	2.83	2.81
	weighted	2.74	2.81	2.82	2.81
	std. dev.	0.07	0.04	0.03	0.00
DFT+D3	Top	2.89	2.97	2.98	3.02
	HCP	2.79	2.92	2.94	3.01
	Bridge	2.76	2.89	2.92	3.04
	mean	2.81	2.93	2.95	3.02
	weighted	2.80	2.92	2.94	3.02
	std. dev.	0.07	0.04	0.03	0.02
DFT+ optB88-VdW	Top	2.85	2.89	2.90	2.90
	HCP	2.64	2.78	2.80	2.90
	Bridge	2.63	2.76	2.79	2.93
	mean	2.71	2.81	2.83	2.91
	weighted	2.68	2.79	2.82	2.91

std. dev.	0.12	0.07	0.06	0.02

Table S5: Fe – molecular ligand bond length from DFT-D2 calculations for NH_3, H_2O and NO trans to NH_3, H_2O, NO and Ag(111). A longer ligand – Fe bond length implies a stronger trans-effect of the trans-ligand.

ligand1/ FePc /ligand2	H_3N-Fe (Å)	O-Fe (Å)	ON-Fe (Å)
(NH_3)/FePc/(NH_3)	2.03	--	--
(H_2O)/FePc/(H_2O)	--	2.04	--
(H_2O)/FePc/(NH_3)	1.99	2.09	--
(NO)/FePc/(NO)	--	--	1.83
(NO)/FePc/(NH_3)	2.16	--	1.74
Ag(111)/FePc/(NH_3)	2.12[a]/2.11[b]	--	--
Ag(111)/FePc/(H_2O)	--	2.40 [a]/2.45[b]	--

[a]*FePc adsorption on Ag with the Fe on top of a surface Ag atom.*

[b]*FePc adsorption on Ag with the Fe on top of a hcp site.*

Charge redistribution maps

The charge redistribution maps, shown in FIG. 2 of the main manuscript and FIG. S2 were calculated by $\Delta\rho = \rho(A+B) - \rho(A) - \rho(B)$, that is, the electron density of the whole system ρ(A+B) minus the one of each component, ρ(A) and ρ(B), with their atomic positions fixed in the optimized structure. For example, the label FePc(H)/Ag + NH3 means Δρ = ρ(NH3/FePc(H)/Ag) - ρ(FePc(H)/Ag) - ρ(NH3), where ρ(NH3/FePc(H)/Ag) is the total electron density of NH3 adsorption on FePc on Ag with the Fe located above the HCP site, and ρ(FePc(H)/Ag) and ρ(NH3) are electron density of FePc(H)/Ag and NH3 with their atoms fixed at the optimized adsorption positions for NH3/FePc(H)/Ag. Thus, Δρ(NH3/FePc(H)/Ag) displays the charge redistribution caused by adsorption of NH3 on FePc/Ag. Similarly, FePc(H)/Ag+H2O shows the charge redistribution induced by H2O adsorption on FePc/Ag.

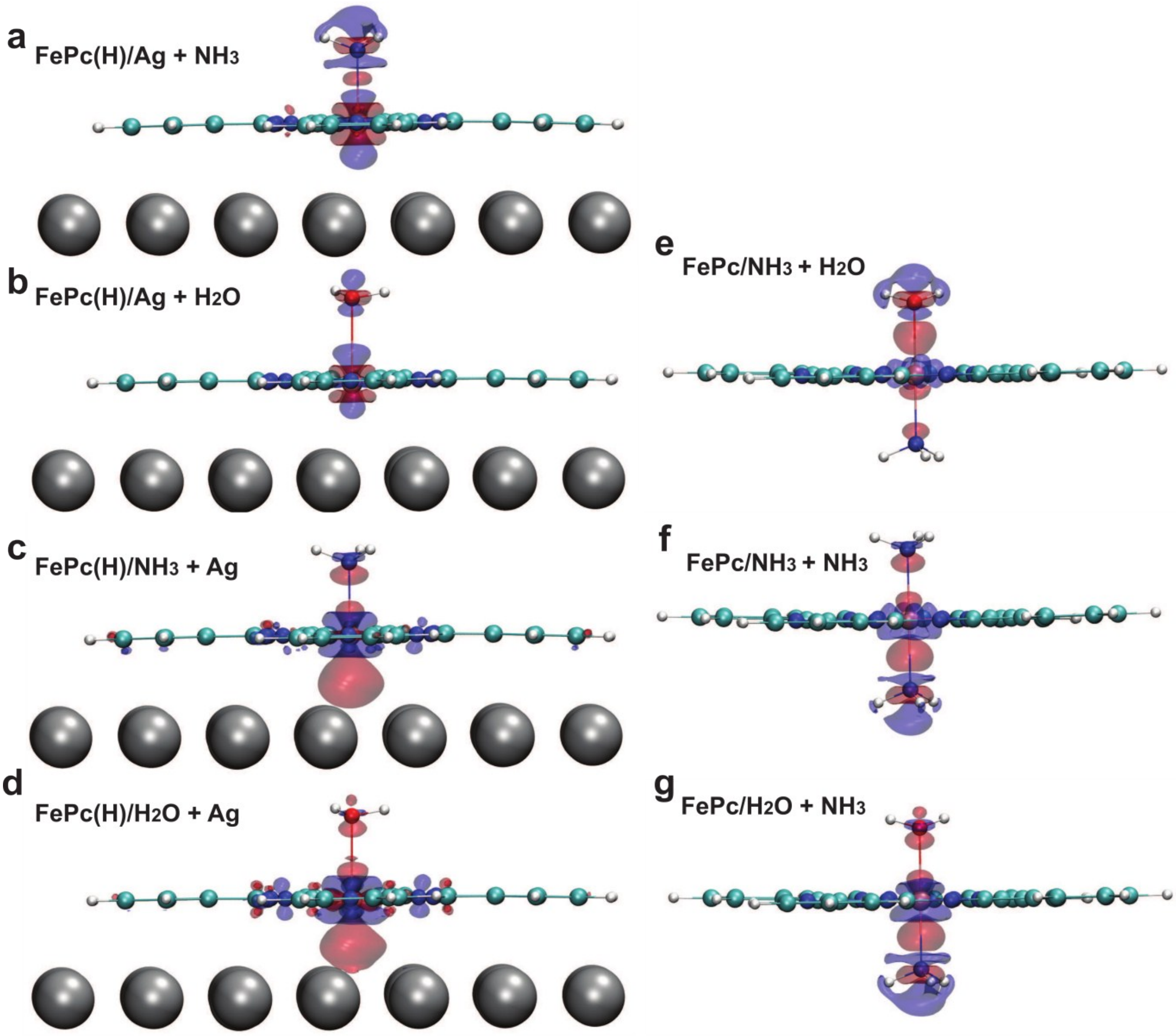

Figure. S2: Calculated charge redistribution maps for introducing (a) ammonia and (b) water trans to the Ag(111) surface; Ag(111) surface trans to (c) ammonia and (d) water; ammonia trans to (g) water and (f) ammonia; and, finally, (e) water trans to ammonia. The charge density difference was plotted at an isosurface of ±0.02 eÅ^{-3}. The red colour represents electron accumulation while blue colour shows electron depletion.

Fig. S2 represents the predicted difference in the local charge density due to the introduction of a given ligand. So, in the case of FIG. S2, the plotted redistribution shows how introducing an ammonia molecule above the Fe atom of FePc adsorbed on Ag(111) results in charge accumulating in the Fe atom's d-xz and d-yz orbitals and a depletion in the Fe atom's d-z^2 orbitals (see panel a).

Electrostatic potential above the surface

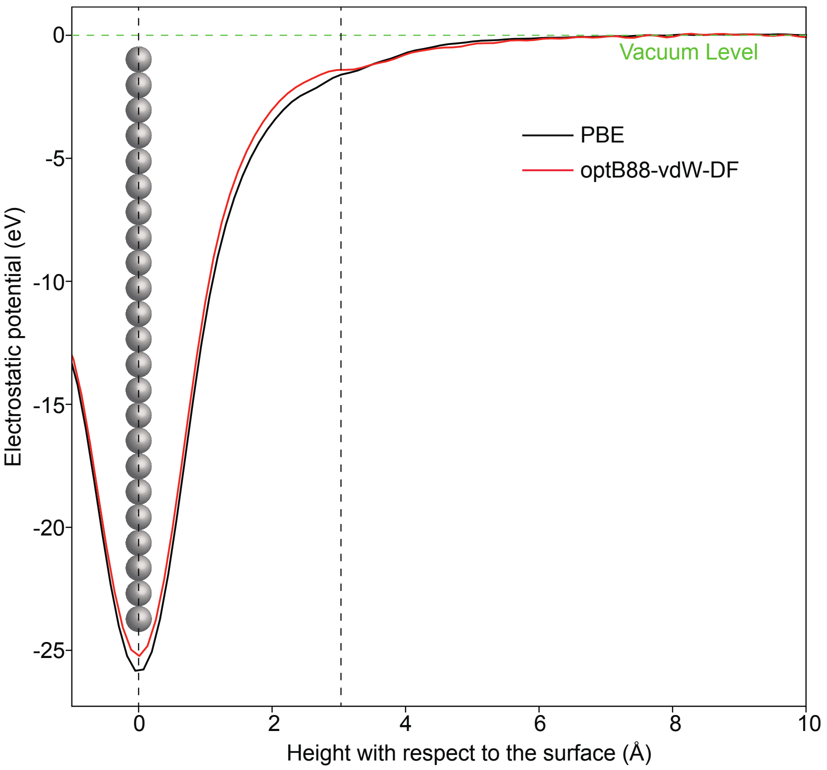

Figure. S3. Calculated plane-averaged electrostatic potential above a clean Ag surface using the PBE functional (black) and the optB88-vdW-DF functional (red). Height of the Ag surface is set to zero. The vacuum level is used as the reference (green dashed line). At the molecular adsorption regime (within 3 Å above the surface, that is between the two dashed lines), optB88-vdW-DF functional calculation shows a less negative potential, corresponding to a lower local work function.

Here we use optB88-vdW-DF calculations to show the calculated electrostatic potential above a clean Ag surface, because calculations using the classical pair-wise force field potential, such as in DFT-D2 and D3 methods, of the same clean Ag surface will show no difference as compared the PBE-functional calculations. Within 3 Å above the surface (between the two black dashed lines), the difference between the electrostatic potential varies by 0.3-0.7 eV, and these two potentials both converge to the vacuum level eventually. The less negative potential of the optB88-vdW-DF calculated Ag surface suggests a lower local work function, corresponding to a different charge distribution at the surface as compared to the PBE-calculated surface, that is a surface dipole pointing towards the surface.

This lower local work function when using vdW-DF functional makes Ag a stronger trans ligand. As shown in Figure 2b, when adding the Ag(111) surface trans to a water molecule, charge accumulation occurs at the interface. The lower local work function (an electric dipole pointing towards the surface) helps such a charge accumulation at the interface, and thus leads to a stronger interfacial binding as compared to the PBE-calculated results.

S1. Isvoranu, C. et al., J. Chem. Phys. 2011, **134**, 114710.
S2. Papageorgiou, N., Ferro, Y., Salomon, E., Allouche, A., Layet, J. M., Giovanelli, L., Le Lay, G., *Phys. Rev. B,* 2003, **68**, 235105
